# “Remember One Size Doesn’t Fit All”: A Scoping Review of Postpartum Supports for Neurodivergent Mothers

**DOI:** 10.1007/s10995-025-04161-z

**Published:** 2025-09-19

**Authors:** Noreen O’Leary, Catherine V. George, Zeinab ElDirani, Ruth Jenks, Gráinne Kent

**Affiliations:** 1 School of Population Health, Royal College of Surgeons in Ireland, 123 St Stephen’s Green, Dublin 2, Ireland; 2 Autistic Parents UK, Birmingham, Great Britain

**Keywords:** Neurodiversity, Scoping review, Postpartum

## Abstract

**Introduction:**

Neurodivergence affects how women experience the world and life transitions such as motherhood and the postpartum period. Postpartum supports are designed from a neurotypical perspective and may not meet the needs of neurodivergent women. For example, breastfeeding groups may not support the sensory needs of autistic women. The aim of this scoping review was to document postpartum experiences of neurodivergent women.

**Methods:**

A scoping review methodology underpinned this review. The socio-ecological model was used to report findings and generate recommendations.

**Results:**

18 records were included primarily representing experiences of autistic women and women with ADHD. Women reported that acting in the best interests of their baby was their highest priority. This often involved making adaptations that disrupted their coping strategies and engaging in social situations such as baby groups, which required them to accept cultural norms and adopt expected neurotypical behaviours. Healthcare professionals did not always account for the needs of neurodivergent women; in some cases, this led to situations whereby neurodivergent women experienced greater parenting scrutiny.

**Discussion:**

This review highlighted a small but growing body of research relating to the postpartum experiences of neurodivergent women. Neurodivergent women need access to tailored supports during the postpartum period as they balance managing the needs of an infant with necessary neurodiversity adjustments. However, there is also a need for greater healthcare professional training specific to supporting neurodivergent women and better public understanding of neurodiversity to ensure neurodivergent women feel safe to be their authentic selves in motherhood.

## Significance

This review highlights the limited research in this area to date, while also casting light on the specific postpartum needs of autistic women and women with ADHD. Many such needs are not recognised or met by current supports. This may lead to women masking neurodivergence and experiencing higher levels of anxiety and burnout, leading to long-term negative outcomes for mother and children. By applying the socio-ecological model we have been able to identify priority actions from the level of individual supports to those needed at a health system and societal level.

## Background

The first 12 months postpartum can be hugely challenging for all women, involving physical and mental recovery from pregnancy and childbirth while caring for a newborn (Madray et al., 2022). For some women there can be additional challenges to navigate. Neurodivergence refers to differences in how individuals process information and experience the world, with up to 20% of the population experiencing neurodivergence (Bell, 2023). Neurodivergence includes dyspraxia, autism, attention deficit hyperactivity disorder and others (Layinka et al., 2024) (Appendix 1).

The postnatal period can highlight specific issues for neurodivergent women. For some, underlying sensory sensitivities may be intensified by increased tactile (e.g. breastfeeding), auditory (e.g. baby cries) and visual (e.g. infant toys) demands (Hampton et al., 2022). It is reasonably well established that social support and connection are key factors in promoting maternal well-being and protecting against postpartum mood disorders such as postpartum depression or anxiety (White et al., 2023). Yet for some neurodivergent women, social communication can be an area of difficulty, making it burdensome to self-advocate to healthcare professionals or engage in traditional forms of social support such as parent or breastfeeding groups (Pohl et al., 2020). Challenges with executive functioning, such as organisation, planning and time management, are common during the postpartum period (Nordenswan et al., 2021) and may be particularly exacerbated for neurodivergent women. Further, societal factors can also increase the risk of mood disorders for neurodivergent women. Neurodivergent women often face greater employment barriers than neurotypical women (Davies et al., 2023) and research has shown that not being in paid employment can increase the risk of postpartum depression and anxiety (Hannon et al., 2023). The cumulative impact of these challenges may place neurodivergent women at increased risk of postpartum mood disorders such as postpartum depression or anxiety (Andersson et al., 2023).

Globally, postpartum services are often limited despite the well-known issues associated with this period (Daly et al., 2022). Research involving women with pre-existing health conditions has focused on postpartum management of physical conditions such as epilepsy (Hope & Harris, 2023), diabetes (Dude et al., 2018) and mental health conditions such as bipolar disorder, affective psychosis, and schizophrenia (Jones et al., 2014). To date, little research or intervention has focused on postpartum experiences of neurodivergent women, who likely require a tailored and inclusive approach which is sensitive to neurobiological differences (Donovan et al., 2023; Stuart & Kitson-Reynolds, 2024). Therefore, the aim of this scoping review was to:

- Summarise what is known about postpartum experiences of neurodivergent women.
- Identify, what, if any, specific postpartum supports exist for neurodivergent women.
- Generate recommendations for tailored postpartum supports for neurodivergent women.

## Method

This scoping review was designed using the Arksey and O’Malley (2005) framework as extended by Levac et al. (2010). The PRISMA-ScR (Preferred Reporting Items for Systematic Reviews and Meta-Analyses Extension for Scoping Reviews) was used to report review processes transparently (Tricco et al., 2018). The protocol was published in Open Science Framework (removed for anonymisation).

### Identifying the Research Question

The key research questions underpinning this scoping review were:

- What is known about postpartum experiences of neurodivergent women?
- What, if any, specific postpartum supports exist for neurodivergent women?
- What is required to develop postpartum supports for neurodivergent wome

###  Identifying Relevant Studies

The following decisions were made prior to commencing the review to balance the comprehensiveness and breadth of the review with available resources.

- All types of empirical research were eligible for inclusion (Randomised controlled trials, cohort studies, case-control studies, cross-sectional studies, qualitative studies) as well as systematic reviews and meta-analyses. Conference proceedings, editorials, commentaries, preprints were also included to ensure any relevant data on this topic was included.
- The following databases were identified as those with greatest relevance to the research question: Embase, Medline, PsycINFO, CINAHL, Scopus, Web of Science, and Cochrane Library.
- Grey literature search included policy documents, dissertations, and working papers produced by government and non-government agencies.
- No date limiters were applied.
- No language limiters were applied.

A specific search string was developed in consultation with a subject librarian to identify relevant studies (Appendix 2). This is based on three concepts related to neurodiversity, postpartum and mental wellbeing. The search was completed on 10th April 2024.

### Study Selection

Empirical data was exported to Rayyan to facilitate data screening and selection. All records were double blind screened by two authors according to the inclusion and exclusion criteria below.

**Table:** 

	Inclusion	Exclusion
(P) Population	● Neurodivergent women who have given birth within the previous 12 months ● Neurodivergent women who are currently pregnant and will have given birth by point of data collection	● Neurodivergent women who have not given birth ● Neurotypical women who have given birth
(O) Outcome	● Postpartum mental wellbeing outcomes for neurodivergent women	● Postpartum outcomes for neurodivergent women other than those related to mental wellbeing. ● Postpartum mental wellbeing outcomes for non-neurodivergent women

### Charting the Data

Data relevant to the research question were extracted and inputted to an Excel spreadsheet which was developed for this review. Headings included: authors, year of publication, country where research was conducted, participants, study design and summary findings.

As this is a scoping review, quality appraisal was not undertaken (Levac et al., 2010).

### Collating, Summarizing, and Reporting Results

Data was analysed using a deductive descriptive approach with a focus on reporting patterns or key information which answered the research questions. Analysis was deductive as it was guided by the Socio-Ecological Framework (SEF). The SEF provides a lens to consider healthcare experiences not only through individual (intrapersonal) factors but also focuses on interpersonal (formal and informal supports), organizational (e.g., healthcare services), environmental (e.g., cultural norms), and policy (maternal healthcare policy) (McLeroy et al., 1988). Analysis was descriptive insofar as it was staying close to the data and minimally interpretive.

### Consultation

Preliminary findings were used as a foundation for stakeholder consultation, which informed refinement of findings.

## Researcher Positionality

Anon 1 is a neurotypical female with experience of working with autistic young people and conducting qualitative research. Anon 2 is as a neurotypical female with experience conducting qualitative research in women and individuals during postpartum period. Anon 3 is a neurotypical female qualitative researcher with a background in public health and experience working on women’s health issues in low- and middle-income countries. Anon 4 is a female with lived experience as an autistic woman with ADHD. Anon 5 is a neurotypical female, with clinical experience as an allied health professional working with neurodivergent adults and young people and in conducting qualitative research.

## Findings

**Fig. 1:**
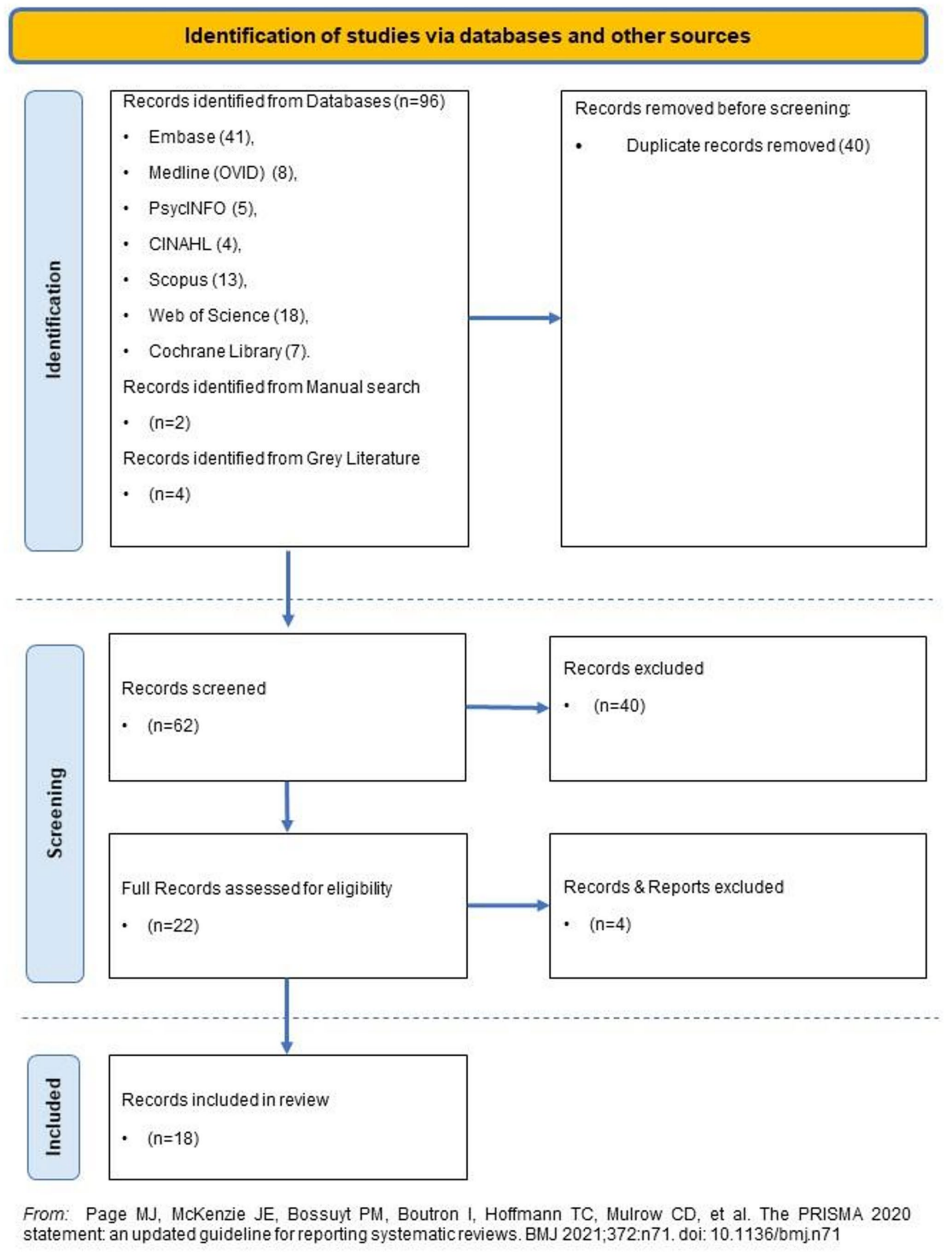
The PRISMA 2020 statement: an updated guideline for reporting systematic reviews (Page et al., 2021)*.*

From a total pool of 102 records, we screened 62 records and 18 met the criteria for inclusion in this scoping review (Fig. 1). A full overview of the 18 included records can be found in Appendix 3. 

We found 12 empirical research articles, one published in 2016, 2019, 2020 and 2021 respectively, three published in 2022 and five published in 2023. One paper was based in Japan, Italy and Sweden respectively, five in the United Kingdom, and three in the USA. One paper contained data from across the United States, the United Kingdom, and Australia. Ten studies focused on autism, one on ADHD and one included spinal cord injury (SCI), traumatic brain injury (TBI) and Autism. Relating to the latter, only the data relating to autism was extracted for the current review. Study designs spanned quantitative, qualitative and mixed methods.

We identified one systematic review from 2022 synthesising data from the United States, United Kingdom and Australia. We also identified a qualitative meta-synthesis from 2023 containing data from studies in Australia, UK, United States, Canada. Both focused on autism.

From the grey literature we found four records, three theses and one commentary paper. The theses were submitted in 2013, 2016 and 2020 respectively. The commentary paper was published in 2022. Two theses focused on autism and one on ADHD. The commentary paper focused on autism. In the next section we will present key findings, organised according to the SEF.

### Intrapersonal Factors

Becoming a mother represented a major life transition for neurodivergent women, as for all women, and, for many, led to an exacerbation of issues specific to neurodivergence. For example, a key issue reported in this area related to sensory challenges associated with autism and high sensory demands involved in the care of young children:

I have trouble with noises and smells and touching and handling other people’s emotional expressions. The “volume” on all these got “louder”. (Autistic mother)

(Hampton, 2020)

Breastfeeding gave rise to particular sensory challenges, with some mothers identifying strategies to help reduce sensory overwhelm:

The hardest thing while I was nursing them was them touching my breasts. From a sensory standpoint, I just can’t stand the way it feels. (Autistic mother)

(Gardner et al., 2016)

I use nipple shields so there’s a little boundary space between me and my baby. It’s not as much of a sensory trigger with the shield. (Autistic mother)

(Wilson & Andrassy, 2022)

The disruption to established routines, often developed as coping mechanisms, was often challenging:

Babies have demanding round-the-clock needs; they are everything that an autistic person would have difficulty coping with. Time alone and carefully crafted routines no longer existed. (Autistic mother)

(Litchman et al., 2019)

It was also clear from the data that mothers were consistently making decisions in the best interests of their child, often to the detriment of their own well-being:

Autistic people aren’t famous for coping well with change, but you know, it is what it is and you just have to kind of adjust as you go and just learn as you go. (Autistic mother)

(Dugdale et al., 2021)

### Interpersonal (Formal and Informal Supports)

The lack of tailored support and understanding from both formal services and personal networks is a significant issue for neurodivergent mothers. This can exacerbate feelings of isolation and overwhelm while navigating new motherhood.

Partner and family understanding of autism improved support availability to mothers:

I can just phone them [husband’s parent] up and say, ‘I need your help, I’m really struggling’. And that’s it, like, they’ll help me. (Autistic mother)

(Dugdale et al., 2021)

Mothers reported finding social supports such as mother and baby groups challenging, as they often felt exposed to judgement of other parents and out of sync with other parents:

I’ve been going to a baby group but I don’t feel like I’ve made much of a connection with anyone. I keep going but I’ve found it really hard. Everyone goes on about how you need a mum network but I don’t have that. (Autistic mother)

(Hampton et al., 2022a, 2022b)

Although they valued being with other mothers, they felt especially incompetent in groups of mothers. Participants reported often feeling “incompetent”, “embarrassed”, “alienated”, and “misunderstood” as a mother as a result of socializing with peers. (Mother with ADHD)

(Curtin-McKenna, 2013)

### Organisational (Healthcare Systems)

Mothers reported that healthcare systems were not designed to provide tailored supports that take diverse needs into account:

You’re all treated exactly the same it’s like a conveyor belt of pregnant women that they just go through one after the other you know, and you get stamped and branded and come out of the other end. (Autistic mother)

(Burton, 2016)

Professionals look at breastfeeding in a very neuro-typically based way. We are all different. Some of us are very dramatic, others are highly logical and literal. Remember one size doesn’t fit all. (Autistic mother)

(Wilson & Andrassy, 2022)

Thus, women felt they were battling the system all the time to get the support they needed; this led to feelings of exhaustion:

You lose your energy your emotions go your energy goes you feel flat in yourself coz you’re just arguing all the time. (Autistic mother)

(Burton, 2016)

Fox (2022) highlighted the anxiety triggered by having multiple professionals involved in the care of the woman and her baby. Therefore, the author argued for the need for a multi-disciplinary approach for women who present with co-occurring and complex clinical presentations. This is especially important because autistic women have much higher risk of many conditions such as obesity, diabetes, epilepsy, cancer, hypertension, etc.

### Environmental (Cultural Norms)

Neurodivergent women often experience pressure to meet societal, cultural, and gendered expectations of mothering, which can negatively impact their psychological well-being. For example, the pressure to conform to neurotypical expectations around parenting, breastfeeding, and social interactions can lead to increased anxiety and stress. Burton et al. 2016 reported that most women in their study felt different to most other mothers. They reported feeling that the expectation was that they would have to adapt and find a way of surviving in a world that did not appreciate diversity:

It was intrusive. People would say, “Don’t you love being a mother?” I didn’t love being a mother … for spectrum women, it’s a nightmare. You’re made to feel ashamed. It’s not that we don’t want to be seen as good parents. It’s just that we want to do it on our own terms. (Autistic mother)

(Gardner et al., 2016)

### Policy (Laws and Policies)

Neurodivergent women felt that policies relating to child welfare and development placed them at greater risk of having their children removed as these policies are predicated on neurotypical parenting and poor understanding of neurodiversity (Hampton et al., 2022):

I thought they were the people who knew best, and they were putting Chloe’s best interests at heart and things like that but as time came on, I thought, well, I’ve done nothing wrong. You’ve got no evidence, so why take her? (Autistic mother)

(Burton, 2016)

The biggest issue occurs when people in authority misinterpret us [women with autism] and call in Child Protective Services, when in reality, nothing is wrong. Ultimately, we are individuals, influenced by being autistic, influenced by being female, but in the end, still individuals. (Autistic mother)

(Litchman et al., 2019)

A very real consequence of this was women delaying or avoiding diagnosis. Autistic women also reported not disclosing their postpartum depression due to the fear of losing custody over their children (Hampton et al., 2020:

It’s easy to cheat the tests they do with questionnaires. I was so worried they would take my son away that I made the form look like I was fine. (Autistic mother)

(Hampton, 2020)

This is particularly significant as quantitative studies included in this review indicated that neurodivergent women are at increased risk of postpartum mood disorders (Hampton et al., 2022a, 2022b; Andersson et al., 2023).

## Discussion

**Fig. 2:**
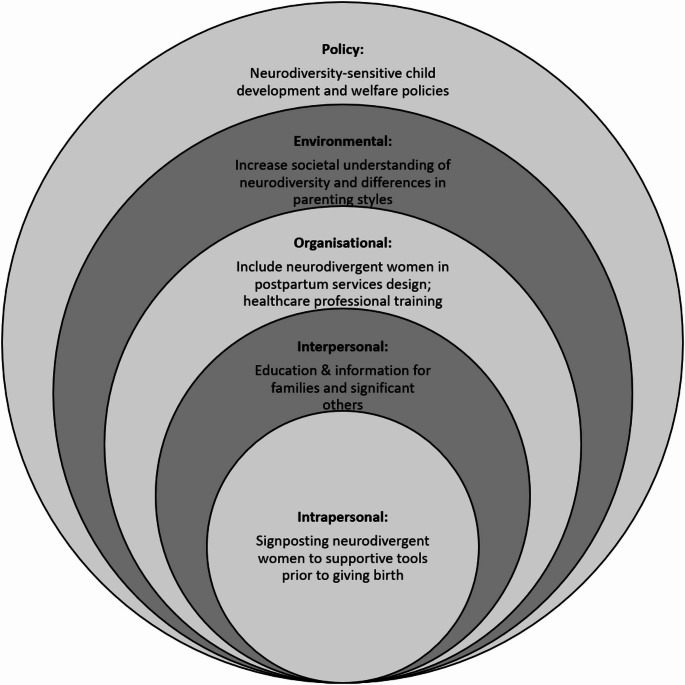
Developing postpartum supports for neurodivergent women

The findings of this review highlighted a small but growing body of research relating to the postpartum experiences of neurodivergent women. In terms of the aims of this review, our findings show that the postpartum period does give rise to specific challenges for neurodivergent women. Currently, there is a paucity of specific postpartum supports for neurodivergent women (Hampton et al., 2023). In this section we integrate current findings with previous literature to generate recommendations for developing postpartum supports for neurodivergent women. This is summarised in Fig. 2 below.

### Intrapersonal Factors

Neurodivergent women may be at increased risk of postpartum mood disorders as they adjust to managing the care of infants with the adaptations required by neurodivergence (Andersson et al., 2023; Hampton et al., 2022b). Signposting pregnant women to tailored information and resources that would help them prepare for this transition may help neurodivergent women prepare for and navigate this period. Recent Danish research has shown that pregnant women do engage with information signposted during antenatal care, with online resources being particularly accessible (Sørensen et al., 2023). However, to be meaningful for neurodivergent women, signposted resources need to be curated. This could be achieved through healthcare provider collaboration with advocacy and support groups.

### Interpersonal Factors

During the postpartum period, women benefit from the social support, including emotional, instrumental (material help) and information support (Sharifipour et al., 2022). During antenatal care, it would be helpful to provide education and support for partners and significant others so they can understand why particular aspects of motherhood may be challenging for neurodivergent women and how they can best provide meaningful support. Neurodivergent people often form relationships with other neurodivergent people (Richards et al., 2022), therefore any resources aimed at partners should also be inclusive of neurodivergent needs. Information support is often obtained through interaction with other mothers at mother and baby groups. As seen in this research these settings can be challenging for neurodivergent women. A useful public health activity may be to develop resources to support the design of inclusive postpartum groups.

### Organisational

It was clear from the reports included in this review that neurodivergent women need postpartum services that are designed and delivered with their specific needs in mind. As we strive to depathologise neurodiversity, resourcing community and peer-led supports would support this and also increase the likelihood of engaging neurodivergent women who may not have had positive experiences of maternal healthcare systems (Rice et al., 2022). Groups developed by neurodivergent people, for neurodivergent people lead to spaces that feel safe and affirming. However, this does not negate the need to improve overall inclusivity of general postpartum services. Indeed, it is imperative that we do not further ‘other’ neurodivergent women whereby they can only access postpartum support through specific groups defined by neurodiversity as all women should be able to access all types of support. It is important that groups and healthcare professionals are inclusive of neurodivergent needs. Including neurodivergent women or advocates in the development of postpartum supports is an important step in achieving this.

Greater awareness among healthcare professionals of what accommodations are needed for neurodivergent women would also improve experiences (Oehme et al., 2024). A key element of this would be to develop healthcare training focused on the interactions between neurodivergent women and healthcare professionals (Ellis et al., 2023), including how to appropriately address potential issues, ensuring neurodivergent women feel safe to share their needs. For example, how to best establish what level of touch an autistic woman is comfortable with during a lactation consultation and ways in which the autistic woman can communicate these needs. Ellis et al. (2023) recommend that to bring about meaningful change, such trainings should be co-designed and delivered by neurodivergent women.

### Environmental (Cultural Norms)

Many women report that they delay diagnosis or do not disclose a diagnosis due to concerns regarding stigma and how their parenting skills will be judged (Thom-Jones et al., 2024). Environmental factors such as cultural norms likely impact this. First, a prevailing culture of infantilization of neurodivergent people and an implicit assumption of reduced parenting capacity (Lo Bosco, 2023). Second, parenting norms are based on neurotypical profiles and may not account for neurodivergent related variations (Thom-Jones et al., 2024). In these cases, women are implicitly having to choose between the impact of non-disclosure and camouflaging their true selves and not being able to ask for or receive support/accommodations that would be helpful. The impact of disclosure can often lead to increased scrutiny of parenting and capacity to parent (Powell et al., 2024).

This cumulative impact of postpartum challenges and camouflaging to meet societal expectations likely places neurodivergent women at increased risk of burnout. Burnout is defined by Raymaker et al. (2020) as pervasive exhaustion, loss of function, and reduced stimulus tolerance due to chronic stress and a mismatch of expectations and abilities without adequate support. Burnout can severely negatively impact parenting ability, leading to long-term negative impacts for both mother and child (Ren et al., 2024). In turn, burnout reduces capacity to mask neurodivergence (Neff, 2025), increasing the likelihood of negative perceptions of parenting. Therefore, at a societal level there is a real and urgent need to improve understanding of neurodiversity and expand our mental models of how parenting may look.

### Implications for Policy

Women have reported distrust of child developmental and welfare systems (Ptacek et al., 2024). Healthcare and child welfare policies continue to be developed in line with neurotypical norms (Hamdan & Bennett, 2024). Such practices may place neurodivergent women at greater risk of appearing to lack parenting skills or capacity, especially during early parenting where we have outlined the specific challenges neurodivergent women may face. During interactions with healthcare professionals, women may not be able to engage as expected (Hwang & Heslop, 2022). For example, autistic mothers have reported increased anxiety when speaking to professionals, to a level where they could not think clearly or express themselves fully (Pohl et al., 2020). This may be taken as evidence of non-engagement or seeking to avoid scrutiny and referral to child welfare services. As such, families may find themselves in circumstances where they are deemed to require parental supervision or fear loss of child custody. The previous recommendations regarding greater neurodiversity training and awareness are further strengthened by these issues.

### Strengths and Limitations

We included a wide range of neurodiversity neurotypes in search terms and did not limit by geographical region. However, the results represented a subset of conditions, primarily autism and minimally ADHD and the experiences of women in a small number of high-income countries. Research needs to consider women with neurotypes such as dyspraxia and traumatic brain injury, as well as the experiences of women in low- and middle-income countries. As this was a scoping review a quality appraisal was not required, as such we cannot comment on or compare study quality. It was not within the scope of this review to consider co-occurrence of included neurotypes. For example, between 50 and 70% of autistic women may also present with ADHD (Hours et al., 2022).

## Conclusions

The postpartum period is a significant life transition for all women and may give rise to specific issues for neurodivergent women. Currently there is a lack of tailored postpartum supports for neurodivergent women. Steps in the right direction would include involving neurodivergent women in the design and development of postpartum supports, specific neurodiversity training for healthcare professionals and increased societal understanding of neurodiversity so neurodivergent women feel better understood and less pressure to camouflage neurodivergence.

## Data Availability

Available from corresponding author on request.

## Appendix

### Appendix 1: Definitions of Neurodiversity

**Table:**

	Definition	Male	Female	Reference
Autism	Characterized by deficits in social communication and the presence of restricted interests and repetitive behaviours	90/10,000	21/10,000	Wang et al. (2022)
ADHD	have signs and symptoms of inattention and/or hyperactivity-impulsivity that make it difficult to function on a day-to-day basis.	177 per 10,000	63 per 10, 000	Cortese (Cortese, 2023)
Developmental Coordination Disorder (DCD)/ dyspraxia	Developmental Coordination Disorder (DCD) is a neurodevelopmental disorder which primarily affects fine and gross motor skills (American Psychiatric Association, 2013).	It is now understood that DCD persists into adulthood in a majority of cases, often creating difficulties in university and work settings; lack of prevalence data about DCD Tal Saban and Kirby (2018)		
Dyscalculia	Dyscalculia is a learning disorder that disrupts math-related skills and abilities. Early treatment can help children learn to adapt to and overcome this disorder.	No prevalence from adults; treatable in children		
Dyslexia	Pronounced difficulties in the selection, timing and spatial organization of purposeful movement and coordination	922 per 10,000	466 per 10,000	Based on a meta-analysis of children by Yang et al. (2022)
Tourette	A neuropsychiatric disorder characterized by multiple motor and vocal tics, lasting at least one year in duration with an onset prior to age 18	1.18 per 10,000		Levine et al. (2019)

### Appendix 2: Search string

**Table:**

Search string	
Key term 1	neurodiversity OR neurodivergent OR neurodivergent OR autism OR asd OR "autism spectrum disorder" OR asperger's OR "asperger's syndrome" OR "autistic disorder" OR aspergers OR adhd OR "attention deficit hyperactivity disorder" OR "attention deficit-hyperactivity disorder" OR dyspraxia OR dcd OR "developmental coordination disorder" OR dyscalculia OR "math disability" OR "mathematics disability" OR "math difficulty" OR "mathematics difficulty" OR dyslexia OR dyslexic OR "reading disability" OR "tourette syndrome" OR "tourette disorder" OR tourettes
Key term 2	postpartum OR post-partum OR post-natal OR postnatal OR "post birth"
Key term 3	"mental wellbeing" OR "psychological wellbeing" OR "emotional wellbeing" OR wellbeing

### Appendix 3: Data Summary

**Table:**

Citation	Year	Country	Diagnosis	Participants	Study design
Empirical research					
Demartini et al. ((2023)	2023	Italy	Autism	33 autistic women without intellectual disabilities; 32 non-autistic mothers	Retrospective observational study
Fukui et al. (2023)	2023	Japan	Autism	2,692 women participated in the study (1263 primipara and 1429 multipara, mean age 31.6 ± 4.8 years)	Cross-sectional
Andersson et al. (2023)	2023	Sweden	ADHD	3515 women with ADHD prior to pregnancy between 2005 and 2013 via the national patient register.	Retrospective population-based review
Dugdale et al. (2021)	2021	UK	Autism	Nine autistic mothers..	Qualitative interview study
Hampton, Man, et al. 2022)	2022	UK	Autism	21 autistic women; 25 non-autistic women.	Qualitative cross-sectional
Talcer et al. (2023)	2023	UK	Autism	7 autistic women	Qualitative interview study
Pohl et al. (2020)	2020	UK	Autism	355 Autistic mothers; 132 non-autistic mothers	Mixed-methods approach
Hampton, Allison, et al. (2022)	2022	UK	Autism	27 autistic women; 25 non-autistic women (third trimester of pregnancy) 24 autistic women; 26 non-autistic women (2 to 3 months after birth) 22 autistic women; 29 non-autistic women (6 months after birth)	Quantitative longitudinal
Donovan et al. (2023)	2023	USA,UK, Australia	Autism	Twenty-four autistic women ages 29–65 years	Qualitative study
Gardner et al. (2016)	2016	USA	Asperger syndrome	8 autistic women	Secondary qualitative analysis
Wilson and Andrassy (2022)	2022	USA	Autism	23 autistic women	Qualitative interview study
Litchman et al. (2019)	2019	USA	SCI, TBI and Autism	1 personal blog (the full data set included 125 blogs; 124 related to SCI and TBI which were not within scope of this review)	Qualitative study
Reviews					
Yau et al. (2023)	2023	USA,UK, Australia	Autism	435 autistic women across 12 peer-reviewed articles	Qualitative Meta-synthesis
Grant et al. (2022)	2022	USA,UK, Australia	Autism	341 participants across eight peer reviewed articles and 14 grey literature pieces	Systematic review
Grey literature					
Burton (2016)	2016	UK	Autism	Seven autistic mothers	PhD Thesis Qualitative study
Curtin-McKenna (2013)	2013	USA	ADHD	65 women (Quantitative); 9 women (Qualitative)	Master’s Thesis Mixed methods
Hampton (2020)	2020	UK	Autism	252 people with a diagnosis of autism;177 people who self-identified as autistic; 551 non-autistic people	Thesis Mixed Methods
Fox (2022)	2022	UK	Autism	Not applicable	Commentary
